# High-Resolution Radar Target Recognition via Inception-Based VGG (IVGG) Networks

**DOI:** 10.1155/2020/8893419

**Published:** 2020-07-18

**Authors:** Wei Wang, Chengwen Zhang, Jinge Tian, Xin Wang, Jianping Ou, Jun Zhang, Ji Li

**Affiliations:** ^1^School of Computer and Communication Engineering, Changsha University of Science and Technology, Changsha 410114, China; ^2^ATR Key Laboratory, National University of Defense Technology, Changsha 410073, China

## Abstract

Aiming at high-resolution radar target recognition, new convolutional neural networks, namely, Inception-based VGG (IVGG) networks, are proposed to classify and recognize different targets in high range resolution profile (HRRP) and synthetic aperture radar (SAR) signals. The IVGG networks have been improved in two aspects. One is to adjust the connection mode of the full connection layer. The other is to introduce the Inception module into the visual geometry group (VGG) network to make the network structure more suik / for radar target recognition. After the Inception module, we also add a point convolutional layer to strengthen the nonlinearity of the network. Compared with the VGG network, IVGG networks are simpler and have fewer parameters. The experiments are compared with GoogLeNet, ResNet18, DenseNet121, and VGG on 4 datasets. The experimental results show that the IVGG networks have better accuracies than the existing convolutional neural networks.

## 1. Introduction

Radar automatic target recognition (RATR) technology can provide inherent characteristics of the target, such as the attributes, categories, and models, and these characteristics can provide richer information for battlefield command decisions. The high-resolution radar echo signal obtained from the wide bandwidth signal transmitted by the broadband radar provides more detailed features of the target, which makes it possible to identify the target type. Therefore, more and more research studies focus on RATR technology.

Traditional methods of radar target automatic recognition include k-nearest neighbor classifier (KNN) and support vector machine learning (SVM) and so on. Zhao and Principe [1] applied support vector machine to automatic target recognition of SAR image. Obozinski et al. [2] proposed the Trace-norm Regularized multitask learning method (TRACE) to solve the problem of recovering a set of common covariates related to several classification problems at the same time. It assumes that all models share a common low-dimensional subspace, but the method cannot be extended to the nonlinear field well. Regularized multitask learning (RMTL) proposed by Evgeniou and Pontil [3] extends the existing kernel-based learning methods of single-task learning, such as SVM. Zhou et al. [4] proposed the clustered multitask learning (CMTL) method to replace multitask learning (MTL). It assumes that multiple tasks follow the cluster structure and achieves high recognition accuracy of SAR image. Zhang and Yeung [5] proposed the multitask relationship learning (MTRL) method, which can learn the correlation between positive and negative tasks autonomously, and the recognition accuracy is higher than that of CMTL. Cong et al. [6] proposed a new classification method by improving MTRL, which can autonomously learn multitask relationship and cluster information of different tasks and be easily expanded to the nonlinear domain. He et al. [7] used the principal component analysis (PCA) method to realize the fast target recognition of SAR image.

With the development of artificial intelligence, more and more applications based on neural networks are used for target recognition [8]. In the field of image target recognition, convolutional neural network (CNN) has achieved great success, which is widely used in object detection and localization, semantic segmentation, target recognition, and so on [9]. Visual geometry group networks (VGGNets) [10] proposed by Simonyan and Zisserman have significantly improved image recognition accuracy by deepening the network depth to 19 layers. In the same year, GoogLeNet [11] proposed by Christian Szegedy used the Inception module to have several parallel convolution routes for extracting input features, which widened the network structure horizontally and deepened the network depth to a certain extent while the network parameters are reduced. Studies have shown that deeper networks have better performance, but deepening the network is faced with the problem of gradient disappearance, and the complex networks also have the risk of overfitting. Residual networks (ResNets) [12] and dense convolutional network (DenseNet) [13, 14] solve the above problems by using skip connections and significantly increase the depth of the network. Recently proposed highway networks, ResNets, and DenseNet have deepened the network structure to more than 100 layers and demonstrated outstanding performance in the field of image recognition.

Different from image data, radar data are sparse and have a little amount. Therefore, the network should be able to extract multidimensional features, and the depth could not be too deep. So, we considered using the Inception module and VGG network for training. VGG networks have limited depth and been proven to have excellent feature extraction capabilities. The Inception module has multipath convolution, which can extract radar multidimensional information for learning, and its internal large-scale convolution kernels are also more effective to extract the information with sparse characteristics. Therefore, we proposed a method to fuse the Inception module with the VGG network.

This paper focuses on target recognition based on 1D HRRP and SAR images and proposes the IVGG convolutional neural network structure which is most suitable for high-resolution radar target recognition. The parameters of IVGG can also be greatly reduced.

## 2. Target Recognition Model: IVGG Networks

### 2.1. VGGNets

VGGNets [10] adopted the convolution filters with a small local receptive field and proposed 6 different network configurations. In VGGNets, the convolution filters are set to 3 × 3 and the max-pooling is 2 × 2, with stride 2.

The contribution of the VGGNet is the application of the 3 × 3 small convolution filters. By stacking small convolution filters, the depth of the network is increased, and the nonlinearity of the convolutional layers is strengthened too [15]. Therefore, the nonlinear function can be better fitted (but the overfitting phenomenon needs to be prevented) and the parameters of the network are reduced.

Before the VGG network was proposed, An et al. also used small convolution filters, but the network was not as deep as VGGNet [16]. The VGGNet has better performance than other convolutional networks in extracting target features.

In the structure of VGGNet, the convolutional layers and pooling layers alternately appear. After two to four convolutional layers, a max-pooling layer is followed. In order to keep the computational complexity of the constituent structures at each feature layer roughly consistent, the number of convolution kernels at the next layer is doubled when the size of the feature map is reduced by half through the max-pooling layer. VGGNet ends with three fully connected layers, which are also the classifier for the system.

### 2.2. The Improved Model: IVGG Network

**Figure 1:**
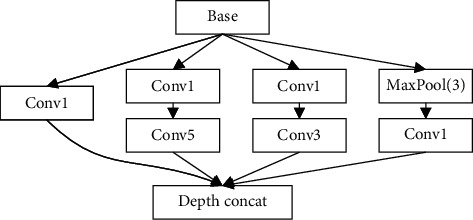
The Inception module, where Conv1 means the convolutional filter is 1 × 1, Conv3 means the convolutional filter is 3 × 3, and Conv5 means the convolutional filter is 5 × 5.

Because SAR images and HRRP data are sparse, it is difficult to fully represent all the feature information of the targets by using all 3 × 3 convolution filters. GoogLeNet, proposed by Christian Szegedy [11], uses the Inception modules with larger convolution filters, which can extract radar multidimensional information for learning. As shown in Figure 1, there are several parallel convolutional lines in the Inception module, and the large convolution filters in parallel lines increase the width and the depth of the network structure. So, the Inception module is used to modify the VGG module. The new network is specially designed for radar data analysis and has a high recognition rate of radar target models. The principle of improvement will be introduced in the next section.

**Figure 2:**
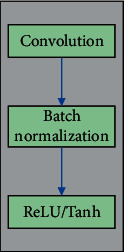
“Conv” module.

In this paper, the “Conv” module includes convolution, batch standardization, and activation functions, as shown in Figure 2.

Based on the above structures, we propose 4 new IVGG networks. In this structure, a certain number of Inception modules are used to replace “Conv3” module in the original VGGNets. Note that we add a very deep point convolutional layer after the Inception module, and it is important. Many traditional algorithms show poor performance for radar target recognition is because they cannot effectively fit the nonlinear structure in the radar signal [6]. Drawing on this point of view, we have strengthened the nonlinear capabilities of IVGG by adding a point convolutional layer. Immediately following the Inception module, the layer contains activation function, which increases the nonlinearity of the network. Further, we set the input number of channels is same with output. In other words, the point convolutional layer does not compress the output feature maps. It also strengthens the nonlinearity of the network.  Table 1 shows the specific configuration of the IVGG networks, where the Inception module and Conv1 module which are used to replace Conv3 modules in the original network are identified in italics.

The fully connected layers of VGGNets are shown in Table 2. Since there have 3 layers, we use “3FC” to refer to the structure in Table 2.

The classifier of the VGG networks is fully connected layers, containing most of the parameters of the whole network. In order to reduce the parameters, we improved the FC layers, reducing the 3-layer FC to a single-layer “FC-4/10”, which is represented by “1FC”.

In the experiment, the network we proposed relates to the above two classifiers, which can be represented by “*IVGGx*-1FC” and “*IVGGx*-3FC,” respectively, where ***x*** is the network depth.

**Figure 3:**
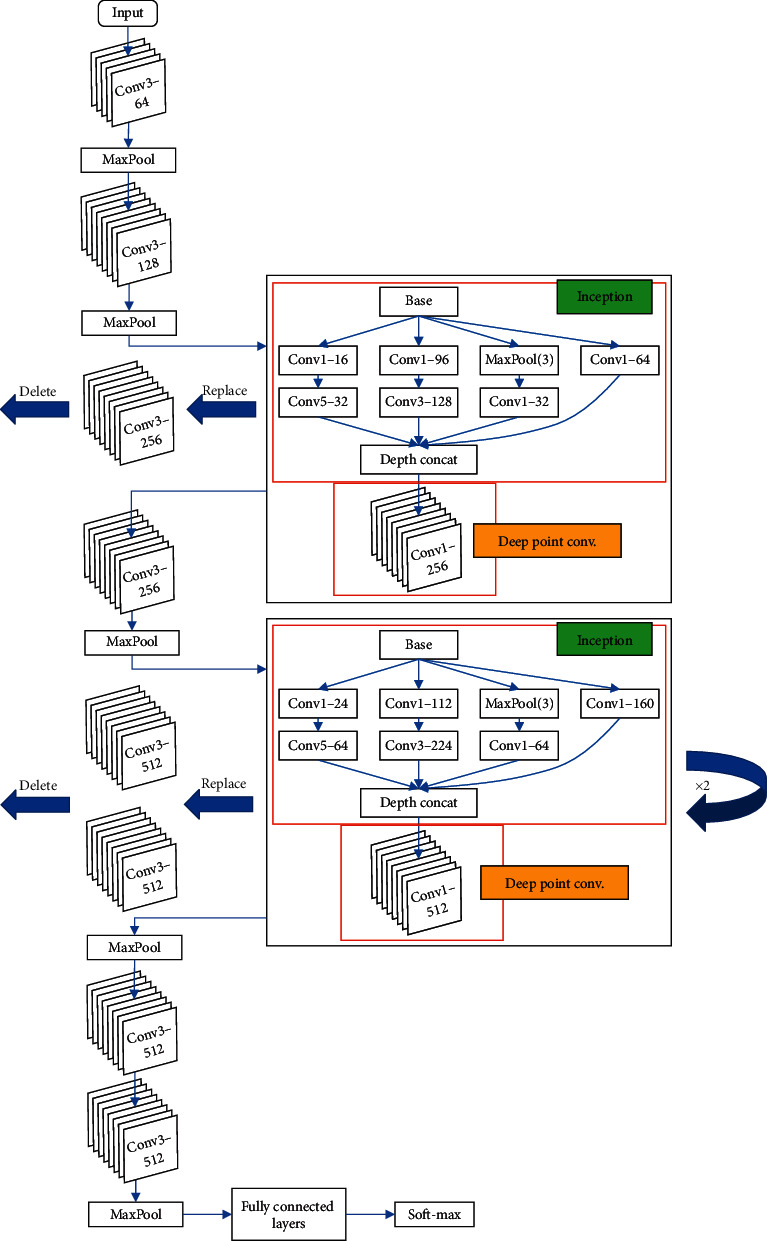
IVGG11 network architecture.

The IVGG11 network is shown in Figure 3, the structure shows how conv3 modules are replaced, and the other networks with different depths (IVGG13/16/19) in Table 1 also follow this rule.

## 3. Characteristic Analysis of IVGG Networks

### 3.1. Relationship between Data Sparsity and Network Structure

In this section, we perform theoretical analysis to demonstrate the sparse characteristics of 3 × 3 filters and 5 × 5 filters. It can further explain that the IVGG network can overcome the target recognition difficulties caused by sparse radar data to some extent.

Assume that in the convolution layers, the weight tensor is *s ***W** ∈ *ℝ*^*C*_in_×*C*_out_×(*k*_1_*k*_2_)^, where *C*_in_ is the number of input channels, *C*_out_ is the number of output channels, and *k*_1_ and *k*_2_ are the convolutional kernel size. Considering the calculation process of convolution filters and feature map in each channel, the weight matrix of the filter is **W**_filter_ ∈ *ℝ*^*k*_1_×*k*_2_^. We unfold the weight matrix into a vector **w** ∈ *ℝ*^*k*_1_*k*_2_^. Each local receptive field in the input (considering a certain channel) is expanded into a vector **x**, and then **w**^*T*^**X** represents the output, where the matrix **X**=(**x**_1_, **x**_2_,…, **x**_*N*_), and the number of elements in the output feature map is represented by *N*.

If the kernel size of a convolution layer is (*k*_1_,  *k*_2_), weight tensor **w**^*T*^=(*w*_1_, *w*_2_,…,*w*_*k*_1_×*k*_2__)^*T*^, the output feature map can be represented as follows:

(1) $$\begin{array}{c} w^{T}X=\left(w^{T}x_{1},w^{T}x_{2},\ldots ,w^{T}x_{N}\right)=\left(\sum_{i=1}^{k_{1}\times k_{2}}w_{i}x_{i,1},\sum_{i=1}^{k_{1}\times k_{2}}w_{i}x_{i,2},\ldots ,\sum_{i=1}^{k_{1}\times k_{2}}w_{i}x_{i,N}\right),\\ w^{T}X_{0}=\#\left(\left.n=\left(1,2,\ldots ,N\right)\right|\sum_{i=1}^{k_{1}\times k_{2}}w_{i}x_{i,n}\neq 0\right),\\ w^{T}X_{1}=\sum_{n=1}^{N}\left|\sum_{i=1}^{k_{1}\times k_{2}}w_{i}x_{i,n}\right|. \end{array}$$

Assume the elements in matrix **X** are set to zero in probability *P*_1_(*P*_1_ < 1), the weight vector **w** element values *w*_*i*_ are set to zero in probability *P*_2_, that is, *P*{*w*_*i*_}=*P*_2_. When *P*_1_⟶1, **X**_0_⟶0, ∀*n*=(1,2,…, *N*), the probability when the neuron is activated is as follows:

(2) $$\begin{array}{c} \underset{P_{1}⟶1}{\lim}P\left\{\overset{5\times 5}{\underset{i=1}{\sum }}w_{i}x_{i,n}\neq 0\right\}=\underset{P_{1}⟶1}{\lim}\left(1-{\left(1-\left(1-P_{1}\right)\left(1-P_{2}\right)\right)}^{25}\right)\\ =\underset{P_{1}⟶1}{\lim}\left(1-{\left(1-\left(1-P_{1}-P_{2}+P_{1}P_{2}\right)\right)}^{25}\right)\\ =1-{\left(1-\left(1-P_{1}-P_{2}+P_{2}\right)\right)}^{25}\\ =1-{\left(1-\left(1-P_{1}\right)\right)}^{25}=1-P_{1}^{25}. \end{array}$$

Similarly, we can get the following expression:

(3) $$\begin{array}{c} \underset{P_{1}⟶1}{\lim}P\left\{\sum_{i=1}^{3\times 3}w_{i}x_{i,n}\neq 0\right\}=1-P_{1}^{9}. \end{array}$$

So, we can get the following inequality:

(4) $$\begin{array}{c} ∴\underset{P_{1}⟶1}{\lim}P\left\{\sum_{i=1}^{5\times 5}w_{i}x_{i,n}\neq 0\right\}>\underset{P_{1}⟶1}{\lim}P\left\{\sum_{i=1}^{3\times 3}w_{i}x_{i,n}\neq 0\right\}. \end{array}$$

When *k*_1_=3, *k*_2_=3, we use *a*_0_ and *a*_1_ to denote **w**^*T*^**X**_0_ and **w**^*T*^**X**_1_. When *k*_1_=5, *k*_2_=5, we use *b*_0_ and *b*_1_  to denote **w**^*T*^**X**_0_ and **w**^*T*^**X**_1_. Then,

(5) $$\begin{array}{c} a_{0}=N\left(1-P_{1}^{9}\right),\\ b_{0}=N\left(1-P_{1}^{25}\right),\\ a_{0}<b_{0}. \end{array}$$

For the convenience of calculations, we assume that input feature vector/tensor does zero padding. Because *P*_1_⟶1, this does not affect the calculation result. Then, we have

(6) $$\begin{array}{c} a_{1}=w^{T}X_{1}=\sum_{n=1}^{N}\left|\sum_{i=1}^{3\times 3}w_{i}x_{i,n}\right|,\\ b_{1}=w^{T}X_{1}=\sum_{n=1}^{N}\left|\sum_{i=1}^{5\times 5}w_{i}x_{i,n}\right|. \end{array}$$

It is easy to prove *a*_1_ < *b*_1_. Therefore, the large-scale convolution kernel can effectively extract the target features if the input data are too sparse.

The sparsity of the convolutional layer can bring many benefits, such as better robustness and higher feature extraction efficiency. However, if the input data are excessive sparse, feature extraction will become more difficult. Therefore, after repeated experiments, we finally chose the Inception module instead of the larger convolution kernel. We just added an appropriate number of Inception module to the network, and they are not all composed of Inception modules like GoogLeNet. In order to improve the network's ability to fit nonlinear structures in radar data (such as SAR images), we add a very deep point convolutional layer behind the Inception module. It should be noted that the point convolutional layer introduces an activation function, and the channels of input and output channels are the same, which improves the nonlinearity of the new network.

### 3.2. The Parameter Number of the Networks

**Figure 4:**
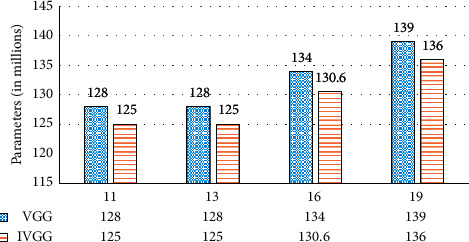
The number of parameters (in millions) of VGG networks and our methods.

As shown in Figure 4, our method has about 3 million parameters less than the VGG network at the same depth. The number of parameters of networks connected to the above two classifiers is shown in Table 3. By improving the classifier, our network can further reduce the parameter amount by 86%–92%.

**Figure 5:**
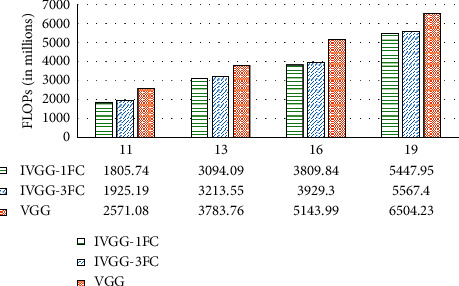
Comparison of floating points of operations (FLOPs).

The comparisons of floating points of operations (FLOPs) are shown in Figure 5. According to Figure 5, the computation cost is most affected by the network depth. IVGG16 and IVGG19 are very computation-intensive. It can be seen from Figure 4 that at the same number of network layers, the FLOPs of IVGG are significantly less than those of the VGG networks. For example, IVGG16-3FC saves 23.61% FLOPs compared to VGG19. So, our methods not only save parameter storage space, but also reduce computation cost.

## 4. Experiment and Results Analysis

### 4.1. Dataset

**Figure 6:**
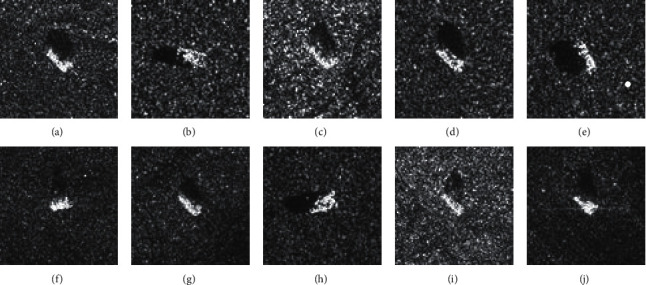
Images of the MSTAR SAR dataset under SOC.

The SAR image dataset used in this paper is a public dataset released by MSTAR. There are many research studies on radar automatic target recognition based on the MATAR SAR dataset, such as references [1–4, 17–20]. The experimental results in this paper are compared with the above methods. The MSTAR dataset and the HRRP dataset are used for experiments. Published by MSTAR [6, 21], the SAR dataset includes ground-based military targets. The acquisition conditions of the MSTAR dataset are classified into standard operating condition (SOC) and extended operating condition (EOC). There are 10 kinds of targets under SOC conditions, each of which contains omnidirectional SAR image data at 15° and 17° pitch angles. In the experiments, observation data at 17° were used for training, and the observation data at 15° pitch angle were used for testing. The optical image of the targets in the MSTAR SAR dataset collected under SOC conditions is shown in Figure 6. In the EOC-1 dataset, there are 4 kinds of ground targets, in which the targets with a side view angle of 17° are used for the training set and the targets with a side view angle of 30° are used for the test set.

The test set and training set are the same model targets in different pitch angles. In fact, this is one of the differences between high-resolution radar target recognition and image recognition. The purpose of this paper is to accurately recognize the target model through high-resolution radar data. In academia, there is only a difference in pitch angle between the test set and the training set, which is feasible and in line with reality [6, 21–23].

Because SAR images are extremely sensitive to changes in pitch angle, it is more difficult to identify the targets under EOC-1 conditions. The pitch angle difference between the SOC training set and the test set is 2°, while the difference under the EOC-1 is increased to 13°. This may lead to a big deviation of the same target in SAR images under the same posture, which increases the difficulty of recognition. Therefore, the experimental conclusions based on the SAR-EOC dataset are more valuable.

As shown in Table 4, the two vectors are two samples in the dataset HRRP-1, which reflects the scattering characteristics of the armored transport vehicle and the heavy transport vehicle, respectively.

The HRRP-1 dataset [22] is target electromagnetic scattering data obtained by high-frequency electromagnetic calculation software. HRRP provides the distribution of target scattering points along the distance and is an important structural feature of the target. HRRP has the characteristics of stable resolution, easy acquisition and realization, and short imaging period. The simulation database contains 4 kinds of ground vehicle targets: armored transport vehicles, heavy transport vehicles, heavy trucks, and vans. Acting on the stepped frequency echo signal at the same observation angle of the target, Inverse fast Fourier transform (IFFT) is used to synthesize the HRRP. Since the electromagnetic simulation data are turntable-like data, it is not necessary to translate and align. In the experiment, the target electromagnetic scattering echo under the HH polarization mode is selected as the basic dataset. The targets with a pitch angle of 27° are used for training, and the targets with a pitch angle of 30° are used for test. Both the training set and the test set have 14400 samples, each of which is a 128 × 1 array with complex data type. The training set is the same as the test set except for the pitch angle. In addition, the HRRP data generated by inversion of the MSTAR SAR dataset are used as the second HRRP dataset (HRRP-2).

### 4.2. Preprocessing and Experimental Setup

For the MSTAR SAR images, each sample is resized to 128 × 128, and then, the center cut and random horizontal rotation are performed. After this preprocessing, the number of SAR images has been expanded by 3 times, which compensates for the shortage of SAR images and alleviates the overfitting problem of the network to some extent.

The phase profile of the complex high-resolution echo of the target can be divided into two parts: the initial phase that is sensitive to the distance and the remaining phase reflecting the scattering characteristics of the target. Therefore, like the amplitude profiles (real HRRP), phase profiles in the complex HRRP also represent a certain information of the scattering point distribution of the target, and it should be valuable in recognition. The complex HRRP contains all the phase information of the target scatter point subecho, including the initial phase and the remaining phase of the scatter point subecho. Therefore, although the complex HRRP has a sensitivity to the initial phase, which is not conducive to HRRP target recognition, it retains other phases information that is helpful for recognition [24]. The traditional RATR uses the amplitude image of HRRP and loses the phase information. Phase information is especially useful for target recognition, but most convolution network models cannot deal with complex data types. At present, the main processing method of complex HRRP is modulus operation, which can keep the amplitude information of range profile and get relatively high recognition accuracy.

Unlike images that can use superresolution method to improve recognition accuracy [25], HRRP is made up of one-dimensional data points, so we propose a new way to preprocess HRRP data. The real part and the imaginary part of each data are extracted and arranged in an orderly way, so that the length of each sample is expanded from 128 to 256. In this way, the differential phase information between the distance units in each HRRP sample can be preserved, and the amount of data in each sample can be expanded.

To compare the test results of different models, the experiments are carried out on the same platform and environment, as shown in Table 5.

Considering that the radar data are sparse, the activation function Rectified Linear Unit (ReLU) [26] will undoubtedly increase this sparseness and reduce the useful information of the target, which is unfavorable for recognition. So, we introduce another activation function, Hyperbolic Tangent function (Tanh). The resulting impact will be further analyzed in the experiments.

The learning rate attenuation method is also introduced in the training processing. As the number of iterations increases, the learning rate gradually decreases. This can ensure that the model does not fluctuate greatly in the later period of training and closer to the optimal solution.

We adjust the parameters according to the results of many experiments and get the final parameters. We use VGGNet pretrained by ImageNet in PyTorch to initialize the parameters of IVGG networks. In the training stage, the batch size of the training set is set to 16 and that of the test set is set to 32. For MSTAR SAR dataset recognition, the initial learning rate is set as 0.01, and 200 epochs are used for training. The learning rate decreases by 2 times since the first 50 epochs and then decreases by 2 times every 20 epochs. The average recognition accuracy of the last 100 epochs was calculated as the final results. For HRRP dataset recognition, the initial learning rate is set as 0.1 and 100 epochs are used for training. The learning rate decreases by 2 times since the first 50 epochs and then decreases by 2 times every 10 epochs. The average recognition accuracy of the last 10 epochs was calculated as the final results.

### 4.3. Recognition Results of the MSTAR SAR Dataset

The recognition accuracy on the MSTAR SAR dataset is shown in Table 6. On SAR-SOC, the results of IVGG networks and VGG networks are better than those of GoogLeNet, ResNet18, and DenseNet121. It can be seen from Table 6 that on the SAR-SOC, IVGG networks with both 1FC and 3FC have good recognition performance. It shows that our methods have better robustness. The recognition rates of IVGG networks are similar to those of VGGNets, but each of them reduces about 3 million parameters compared with the latter.

GoogLeNet achieves high recognition accuracies on SAR-SOC, but its recognition accuracies on SAR-EOC-1 are poor, which are only 90.62% and 90.19%. This shows that its generalization ability is not so ideal. Based on the horizontal comparison of the recognition accuracies of the activation functions, Tanh and ReLU in Table 6, we can see the performance of Tanh on SAR-EOC-1 is generally stronger, indicating that Tanh has a better effect on sparse data processing.

On SAR-SOC, IVGG16-3FC with “Tanh” achieves a maximum accuracy of 99.51%. On SAR-EOC-1, IVGG19-3FC achieves the highest accuracy of 99.27%, and IVGG13-3/1FC also achieves the accuracy of 99.22%. The classification on SAR-EOC is more difficult, and it requires that CNNs have higher performance. So, we especially focus on analyzing the experimental results on SAR-EOC.

The accuracy rate of IVGG13 on SAR-EOC is significantly higher than those of GoogLeNet, ResNet18, DenseNet121, VGG11, and VGG13. It is still 0.12% higher than VGG16 and VGG19. But the parameter number of IVGG13 is only 4.45% of VGG16 and 4.29% of VGG19, and the FLOPs are significantly lower than those of VGG16 and VGG19. Specifically, IVGG13-1FC saves 39.85% FLOPs than VGG16 and 52.43% than VGG19. The accuracy rate of IVGG13-1/3FC is only 0.05% lower than that of IVGG19-3FC, but the parameter of IVGG13-1FC is only 4.77% of that of IVGG19-3FC and the FLOPs of IVGG13-1/3FC are only about 56% of those of IVGG19-3FC.

The experiments show that the IVGG networks can work well on the SAR image public dataset and have good robustness and recognition performance. The important point is that IVGG uses a significantly shallower network to achieve better accuracy than other CNNs. It greatly improves the computational efficiency and can save great parameter space. In fact, IVGG13-1FC relies on relatively less parameters and FLPOs to achieve quite good results. In contrast, although IVGG16 and IVGG19 networks can slightly improve the recognition accuracy, they have paid a high price (increase in parameters and computational cost). We further compare the experimental results of the IVGG13-1FC network with other deep learning methods, proposed by Wang et al. [17], Pei et al. [18], and Chen et al. [19], as shown in Table 7. These literature studies use the same SAR image dataset with this paper. Wang et al. [17] proposed a method for SAR images target recognition by combining two-dimensional principal component analysis (2DPCA) and L2 regularization constraint stochastic configuration network (SCN). They applied the 2DPCA method to extract the features of SAR images. By combining 2DPCA and SCN (random learning model with a single hidden layer), the 2DPCA-SCN algorithm achieved good performance. Due to the limited original SAR images, it is difficult to effectively train the neural networks. To solve this problem, Pei et al. [18] proposed a multiview deep neural network. This deep neural network includes a parallel network topology with multiple inputs, which can learn the features of SAR images with different views layer by layer. Chen et al. [19] used all convolutional neural networks (A-CNNs) [27] to the target recognition of SAR images. Under the standard operating condition, the recognition accuracy on the SAR-SOC image dataset is remarkably high, but the recognition accuracy has declined under extended operating condition.

Although some methods such as A-CNN can achieve accuracy of 99.41% on the SAR-SOC, it is difficult to achieve satisfactory results on SAR-EOC-1 data which have a greater difference in pitch angles. The 2DPCA-SCN method achieves 98.49% accuracy on SAR-EOC-1, but only 95.80% on SAR-SOC. Other methods on the SAR-EOC-1 also achieve lower recognition accuracies than our methods. It can be found from Table 6 that IVGG networks achieve exceedingly high accuracies on both SAR-SOC and SAR-EOC-1 datasets. In particular, on the SAR-EOC-1 dataset, IVGG13 can achieve higher accuracy and more stable performance, which shows that our network has stronger generalization ability and better robustness.

IVGG13-1FC is also compared with traditional recognition methods such as KNN, SVM, and SRC [6, 23], and the results are shown in Table 8. The method proposed in reference [6] is a new classification approach of clustering multitask learning theory (I-CMTL), and SRC is a recognition method based on sparse representation-based classifier (SRC) proposed in 2016 [23]. From Table 8, we can see that our network is better than those of all the traditional recognition methods.

Table 8 shows that some traditional approaches are not so effective, such as KNN and SVM methods. Although many complex classifiers have been designed, they cannot fully utilize the potential correlation between multiple radar categories. On the other hand, large-scale and complete SAR datasets are difficult to collect, so the samples obtained are usually limited or unbalanced.

The classification algorithm approaches under the multitask framework have higher recognition accuracies, such as CMTL, MTRL, and I-MTRL. The multitask relational learning (MTRL) method proposed in [6] can autonomously learn the correlation between positive and negative tasks, and it can be easily extended to the nonlinear field. The MTRL is further improved by adding a projection regularization term to the objective function [7], which can independently learn multitask relationships and cluster information of different tasks and can also be easily extended to the nonlinear field. However, the Trace-norm Regularized multitask learning (TRACE), which is also under the multitask framework, has the lowest recognition accuracy because the TRACE method learns the linear prediction function and cannot accurately describe the nonlinear structure of SAR image, which also proves the importance of extending the multitask learning method to the nonlinear field.

The IVGG networks proposed in this paper can adaptively learn the nonlinear structure of SAR images and reduce the difficulty in redesigning the classifier when the SAR image conditions change. In contrast, the artificially designed feature extraction approach is complex, and sometimes, it can only be effective for certain fixed problems. Its generalization ability is not so ideal. Therefore, our networks enhance the feature extraction capability of sparse data.

### 4.4. Recognition Result of the HRRP Dataset

The recognition accuracy rates on the HRRP dataset are shown in Table 9

On the HRRP-1 dataset, the optimal recognition accuracies of GoogLeNet, ResNet18, and DenseNet121 are 98.7132%, 98.5234%, and 98.7299%, respectively, and the performance of the activation function Tanh is slightly better than that of ReLU. The best recognition results (accuracy > 99.05%) are all obtained by the activation function Tanh. The networks with recognition rate higher than 99.05% are VGG13 (Tanh), IVGG16-3FC (Tanh), and IVGG19-3FC (Tanh). Among them, the recognition rate of IVGG16-3FC (Tanh) is the highest, reaching 99.24%.

In the identification of the HRRP-1 dataset, the networks which are deeper have better recognition results. IVGG16 and IVGG19 can achieve better recognition effects.

The network with the best recognition accuracy on the HRRP-2 dataset is IVGG19-3FC (ReLU). The VGGNet and IVGG-3FC have higher recognition accuracies. The recognition results of IVGG networks and VGGNets have no obvious difference, among which IVGG19-3FC (ReLU) achieves the best recognition accuracy of 98.98%.

On the HRRP-1 dataset, our method is also compared with other methods such as SVM, Maximum Correlation Criterion-Template Matching Method (MCC-TMM) [28], Bayesian Compressive Sensing (BCS) [29], Joint Sparse Representation (JSR) [30], and a CNN method with SVM as its classifier [20], as shown in Table 10.

### 4.5. Comprehensive Analysis of Results

In conclusion, we find that DenseNet121 also has high performance in the SAR dataset (still slightly inferior to our method), but its recognition performance for HRRP is obviously reduced. In HRRP recognition, ResNet18 has a high performance (still slightly inferior to our method), but the performance of SAR image recognition is exceptionally low (only 80%). Different from the above two methods, our method has high recognition performance for SAR and HRRP signals, which means that the method in this paper is efficient and stable. VGG network achieves good performance for radar target recognition, but IVGG reduces the parameters significantly and improves the computation and recognition efficiency.

The performances of IVGG networks are better than those of VGGNets on the HRRP-1 dataset and SAR-EOC-1 dataset and better than those of other neural networks and traditional algorithms on all the experimental datasets.

In fact, the SAR image dataset used in this paper is a public dataset published by MSTAR, and the HRRP dataset also has been published in other papers. The radar is sensitive to the pitch angles, and the radar echo data of the same target at different pitch angles are quite different. This is also the difficulty of radar target recognition. On the SAR-EOC dataset, the difference of pitch angles between the test set and the training set is greater than that on SAR-SOC, and the recognition accuracy on the SAR-EOC test set is slightly lower than that on SAR-SOC.

In addition, we also found a problem in the experiment. When the network comes very deep, the recognition algorithm may be invalid. For example, when we use ResNet50, it will cause the method loss efficacy. The reason is that the data amount of each sample is small (especially HRRP is one-dimensional data), and the downsampling layers in the ResNet50 are too many for HRRP. This problem may also occur in SAR images. But overall, SAR images will be slightly better. Solving this problem has two points, one feasible method is to reduce the downsampling layers, but it will undoubtedly weaken the robustness of the network, which may lead to insufficient results and waste in computing costs. Another effective solution is to design shallow convolutional neural networks for radar target recognition, such as the IVGG networks proposed in this paper.

For target recognition in radar signals, the IVGG networks and VGGNets perform better than several convolutional neural networks recently proposed. The main reasons are as follows.

The noise of the optical image is usually additive noise, while the noise of the SAR image is mostly speckled multiplicative noise. HRRP data are a one-dimensional array, which is the vector sum of projection of the target scattering point echoes in the radar ray direction. Neither of them has obvious edge features and texture information like the traditional optical image. SAR image is sensitive to the azimuth of the target when it is imaged. When the azimuth is different, even for the same target, there are still excessively big differences in SAR images.

The data amount of HRRP and SAR images is less than that of traditional optical images. In this paper, only 256 data per HRRP target and 128 × 128 = 16384 data per SAR image are sent into the networks. However, a slightly larger optical image can often reach 256 × 256=65536 pixels. For this reason, the CNN models for radar target recognition cannot be too deep. Otherwise, they may fall into overfitting. So, compared with ResNet and DenseNet, IVGG networks and VGGNets with fewer network layers have better recognition ability.

In the experiment, the activation function Tanh has excellent performance on the SAR-EOC-1 and HRRP datasets. The radar data itself have sparsity, which is enhanced by the activation function ReLU, while too sparse data will weaken the ability of the convolution layer to extract target features. Activation function Tanh has better nonlinearity and works better when the feature difference is obvious.

## 5. Conclusion

In this paper, we propose the IVGG networks and use them for target recognition on HRRP data and SAR images. The first improvement in this paper is to propose the IVGG networks. Then we simplify the fully connected layers which can significantly reduce parameters. Experiments show that our methods have the best recognition effect. At the same time, with the improvement of the networks, there are fewer parameters in the networks, which can improve the processing efficiency of target recognition and make the method more suitable for the real-time requirements.

In addition, we also find that for radar target recognition, Tanh's performance is generally better than that of ReLU, which is different from image recognition.

## Figures and Tables

**Table 1:** IVGG network configuration.

IVGG11	IVGG13	IVGG16	IVGG19
11 weight layers	13 weight layers	16 weight layers	19 weight layers

Input (HRRP OR SAR)			
conv3-64	conv3-64	conv3-64	conv3-64
	conv3-64	conv3-64	conv3-64

MaxPool			
conv3-128	conv3-128	conv3-128	conv3-128
	conv3-128	conv3-128	conv3-128

MaxPool			
*Inception-256*	conv3-256	conv3-256	conv3-256
*conv1-256*	*Inception-256*	*Inception-256*	*Inception-256*
conv3-256	*conv1-256*	*conv1-256*	*conv1-256*
		conv3-256	conv3-256
			conv3-256

MaxPool			
*Inception-512*	conv3-512	conv3-512	*Inception-512*
*conv1-512*	conv3-512	*Inception-512*	*conv1-512*
*Inception-512*		*conv1-512*	conv3-512
*conv1-512*		*Inception-512*	conv3-512
		*conv1-512*	*Inception-512*
			*conv1-512*

MaxPool			
conv3-512	*Inception-512*	conv3-512	conv3-512
conv3-512	*conv1-512*	conv3-512	conv3-512
	*Inception-512*	conv3-512	conv3-512
	*conv1-512*		conv3-512

MaxPool			

Fully connected layers			

Soft-max			

**Table 2:** Three fully connected layers (3FC).

*FC-4096*
*FC-4096*
*FC-4/10*

**Table 3:** The number of parameters (in millions) of our networks with different classifiers.

Network	1FC	3FC
IVGG11	7.19	125
IVGG13	5.96	125
IVGG16	11.27	130.6
IVGG19	17.67	136

**Table 4:** The samples of complex HRRP vector.

Sample 1 of HRRP	Sample 2 of HRRP
5.947548139439314*e* − 04–7.029982346588466*e* − 04*i*	−0.001741710511154 + 0.005854695561424*i*
5.973508449729275*e* − 04–7.301167648045039*e* − 04*i*	−0.001602329272711 + 0.005996485005943*i*
5.998884995750467*e* − 04–7.586149497061626*e* − 04*i*	−0.001459788439038 + 0.006143776077643*i*
6.023640017197894*e* − 04–7.885879483632503*e* − 04*i*	−0.001313674253423 + 0.006297298858010*i*
6.047727981516010*e* − 04–8.201413412111810*e* − 04*i*	−0.001163535049426 + 0.006457875798999*i*
…	…

**Table 5:** Experimental platform configuration.

Attribute	Configuration information
OS	Ubuntu 14.04.5 LTS
CPU	Intel (R) Xeon (R) CPU E5-2670 v3 @ 2.30 GHz
GPU	GeForce GTX TITAN X
CUDNN	CUDNN 6.0.21
CUDA	CUDA 8.0.61
Framework	PyTorch

**Table 6:** Accuracy rates (%) on the MSTAR SAR dataset.

Method	SAR-SOC		SAR-EOC-1	
	Tanh	ReLU	Tanh	ReLU
GoogLeNet	98.87	98.65	90.62	90.19
ResNet18	97.20	97.90	78.45	82.25
DenseNet121 (*k* = 32)	98.66	98.93	96.41	98.66
VGG11	99.31	99.32	98.61	97.60
VGG13	99.22	99.48	98.22	97.54
VGG16	99.14	99.50	99.10	96.75
VGG19	99.26	99.21	99.10	97.91
IVGG11-3FC	99.21	98.98	97.97	98.05
IVGG11-1FC	99.23	99.13	97.02	97.73
IVGG13-3FC	99.04	99.31	**99.22**	98.04
IVGG13-1FC	99.34	99.14	**99.22**	98.24
IVGG16-3FC	**99.51**	99.34	98.84	98.70
IVGG16-1FC	**99.42**	99.19	97.62	97.68
IVGG19-3FC	99.42	99.23	**99.27**	97.71
IVGG19-1FC	99.23	**99.37**	97.15	98.47

**Table 7:** Accuracy rates (%) on the MSTAR SAR dataset of different CNNs.

Method	SAR-SOC	SAR-EOC-1
2DPCA-SCN [17]	95.80	98.49
2-view DCNNs [18]	97.81	93.29
3-view DCNNs [18]	98.17	94.34
4-view DCNNs [18]	98.52	94.61
A-CNN [19]	**99.41**	97.13
IVGG13-1FC	**99.34**	**99.22**

**Table 8:** Accuracy rates (%) of different methods on the SAR dataset.

Method	SAR-SOC	SAR-EOC-1
KNN [1]	92.71	91.42
SVM [1]	90.17	86.73
SRC [23]	89.76	—
TRACE [2]	75.04	67.42
RMTL [3]	92.09	92.03
CMTL [4]	93.91	94.72
MTRL [5]	95.84	95.46
I-CMTL [6]	97.34	98.24
**IVGG13-1FC**	**99.34**	**99.22**

**Table 9:** Accuracy rates (%) on the HRRP dataset.

Method	HRRP-1		HRRP-2	
	Tanh	ReLU	Tanh	ReLU
GoogLeNet	98.71	97.95	98.48	97.85
ResNet18	98.52	98.02	98.48	98.20
DenseNet121	98.73	97.94	98.15	97.65
VGG11	98.32	98.18	98.56	97.51
VGG13	99.05	98.89	98.76	98.79
VGG16	98.75	98.55	98.94	98.88
VGG19	98.90	98.40	98.66	98.76
IVGG11-3FC	98.79	97.76	98.35	98.19
IVGG11-1FC	98.52	98.28	97.95	98.42
IVGG13-3FC	98.75	98.86	98.65	98.80
IVGG13-1FC	98.46	98.43	98.33	98.54
IVGG16-3FC	**99.24**	98.99	**98.90**	98.67
IVGG16-1FC	98.54	98.79	98.50	98.63
IVGG19-3FC	**99.06**	99.05	**98.84**	**98.98**
IVGG19-1FC	98.35	98.84	98.02	98.11

**Table 10:** Accuracy rates (%) of different methods on the HRRP-1 dataset.

Method	Accuracy rate (%)
SVCA + SVM [28]	94.24
MCC-TMM [28]	92.81
BCS [29]	92.76
JSR [30]	91.49
CNN + SVM [20]	96.45
IVGG16-3FC	**99.24**

## Data Availability

All datasets in this article are public datasets and can be found on public websites.
